# The dual effect of vagus nerve stimulation in pediatric patients with drug-resistant epilepsy: Is there more than seizure control?

**DOI:** 10.1016/j.ebr.2024.100653

**Published:** 2024-02-12

**Authors:** Mohamed Ashraf Mahmoud, Omnia El Rashidi, George Halim, Mohamed Amgad Elkholy, Osama Aglan, Abdel Rahman El Sabbagh, Ahmed Kamel Basha, Hussein Hamdi, Ahmed M. El Sayed, Dina Amin Saleh, R.H. Shatla, Walid Abdel Ghany

**Affiliations:** a Neurosurgery Department, Ain Shams University, Cairo, Egypt; b Department of Pediatrics, Ain Shams University, Cairo, Egypt; c Neurosurgery Department, Tanta University, Tanta, Egypt

**Keywords:** Cognitive, Drug-resistant epilepsy, Neuromodulation, Seizure, Vagus nerve stimulation, Cerebral Palsy

## Abstract

This is a retrospective and comparative pilot study to investigate the role of vagus nerve stimulation (VNS) in improving cognitive functions in the pediatric age group with drug resistant epilepsy (DRE). It was conducted from January 2018 to February 2023. Children between the ages of 4 and 18 years were divided into two groups, the “VNS group” and the “best medical treatment (BMT) group”. Follow up period was 12 months. Demographic, clinical, etiological and investigational data were recorded. Cognitive assessment using the Modified Mini-Mental State Examination for children (MMSE) was recorded at baseline and 12 months later for each group. 76.4 % of patients were classified as epilepsy secondary to cerebral palsy. 75 % of patients showed ≥ 50 % seizure frequency reduction among the VNS group as compared to 12.5 % in the BMT group. None of both groups achieved seizure freedom. At 12 months, both BMT and VNS groups showed statistically significantly improved overall cognitive score from baseline records (p = 0.027) and (p = 0.012), respectively, with a significantly higher improvement in VNS group. Also, statistical sub-analysis of cognitive subscales in cerebral palsy patients in both groups was conducted and revealed a significant improvement (p = 0.02) in the VNS group. We concluded that there is a potential role of VNS in improving cognitive functions which was shown by using a cost-effective screening tool. A significant effect was observed specially in cerebral palsy patients. This is very beneficial in limited-resources countries since VNS has good safety profile, high seizure control, and added value to cognitive functions.

## Highlights

• Vagal nerve stimulation (VNS) is used in Drug-resistant epilepsy (DRE) pediatric patients not amenable to resective surgery.
• VNS showed effective seizure control compared to best medical treatment (BMT)at 12 months follow up.
• Both BMT and VNS showed significant improvement in overall cognitive scores, with higher scores in the VNS group.
• Sub-analysis of cerebral palsy patients showed significant improvement in cognitive scores with VNS.
• VNS very beneficial in limited-resources countries with high safety profile and high economic value in the long term.

## Introduction

Epilepsy is a chronic debilitating brain disorder that affects millions of patients worldwide, of which approximately 10.5 million are estimated to be in the pediatric age group [1], [2]. The median lifetime prevalence of epilepsy in the Arab world was estimated to be 6.9 per 1000, however, epidemiological studies were lacking in half of the Arab countries [3]. Epilepsy constitutes a public health burden, where one-third of patients will still suffer from drug-resistant epilepsy (DRE) despite the ongoing innovation in pharmacological therapy [4]. This is more pronounced in low/middle-income countries leading to a reduction of the quality of life (QOL) [5] which can be attributed to limited resources, parental consanguinity, family history of epilepsy, poor antenatal care, and lack of disease awareness [3].

In 2017, vagal nerve stimulation (VNS) was granted Food and Drug Administration (FDA) approval as a novel neuromodulation procedure for the treatment of seizures in pediatric patients with DRE who are not candidates for resection or ablative surgery [6]. It is well known that DRE is commonly linked to neuropsychological impairment in children and may disrupt their social development [7]. Recent literature suggests that VNS might have a supplementary role in refining the cognitive functions, mood, and the QOL of patients with DRE [8], [9], [10]. Several explanations were proposed for cognitive improvement, such as improvement in sleep patterns hence improving consolidation of learned information and QOL, increase in alertness and learning process, and suppression of interictal epileptiform discharges (IED) that are widely accepted to contribute to neurocognitive impairment [11].

All of the published literature supporting this concept comes from the developed regions. We believe that we have unique socio-demographic/cultural differences in our region that warrant investigating similar concepts. Therefore, we aimed at assessing the cognitive functions at baseline and after 12 months in patients who underwent VNS implantation versus those who received the best medical therapy (BMT). During this short period, it is unlikely that any cognitive improvement could be attributed only to the natural course of brain development or improvement in the learning process. It is of great importance to delineate the extent of cognitive improvement in pediatric patients with DRE irrespective of their underlying structural brain pathology since it has a huge impact on the mental growth of children, as opposed to adults in whom further mental growth is not expected.

## Methods

### Study Design

This retrospective comparative study included 17 pediatric patients with DRE who visited our Functional Neurosurgery clinic at Ain Shams University Hospital for evaluation during the period between January 2018 and February 2023. They failed to respond to at least three properly chosen anti-seizure medications (ASMs), ketogenic diet, and were not amenable or refusing resective epilepsy surgery. Two groups of patients were identified, the “VNS group” who underwent VNS implantation, and the “BMT group” who were either eligible and did not have access to VNS implantation or were not willing to proceed with VNS implantation. Demographic data including “biological sex, age on presentation, age at onset of seizures, age at VNS implantation for the VNS group”, detailed neurological examination, and EEG findings were recorded. All patient's charts were reviewed at baseline for epilepsy characteristics, the most disabling seizure type, mean seizure frequency over a one-month period (obtained from seizure diaries kept by the caregivers), seizure duration, and the number of current ASMs. Patients with DRE who failed to complete a follow-up period of 12 months were excluded from the study.

Inclusion criteria

• Age 4–18 years
• Participant not amenable for resective epilepsy procedures.
• EEG shows multifocal epileptiform discharges.
• Children not controlled with three or more ASMs.

Exclusion criteria

• Children who missed follow-up visits.
• Children who had previous epilepsy surgery.
• Deteriorating neurologic or medical condition.
• Children unfit for surgery.
• Non-epileptic seizures.

### VNS surgical procedure

#### Pre-operative preparations

All patients who were eligible for VNS implantation underwent preoperative evaluation which included detailed analysis of the seizure semiology, long-term video Electroencephalography (EEG) monitoring, and brain Magnetic Resonance Imaging (MRI) examination. Also, full laboratory workup, echocardiography, and neck ultrasound to exclude any structural neck anomalies or aberrant vasculature were performed [12]. All children received prophylactic double antibiotics (anti-Gram + ve and anti-Gram −ve) as per our hospital protocol (Ampicillin-sulbactam 50 mg/kg and Ceftriaxone 50 mg/kg).

#### Intra-operative and operative technique

**Fig. 1:**
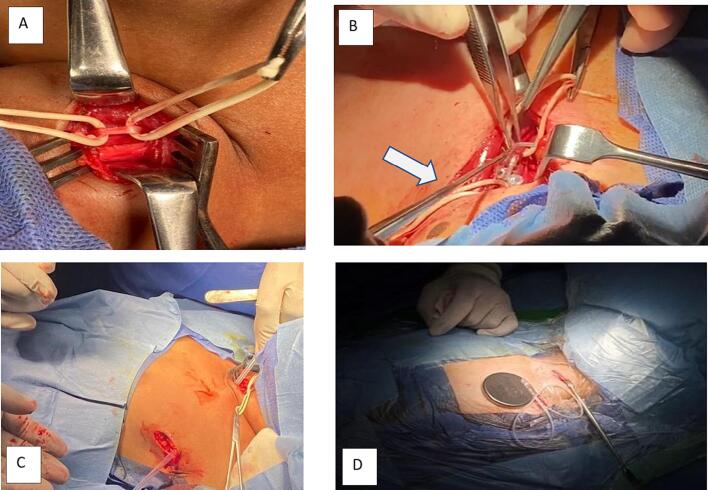
Operative steps of VNS device implantation**(A)**Left Vagus nerve dissection in the carotid sheath and elevation with rubber slings. **(B)** Coiling process of the Vagus nerve using forceps and cervical nerve hooks (white arrow) to ease coiling of wire loops around the nerve. **(C)** Tunneling of the wires from coils to battery via anterior axillary fold incision. **(D)** Battery implantation in the created pocket.

Aspire SR device (model 106) manufactured by Liva Nova − London, United Kingdom was implanted [13]. Device implantation was done under general anesthesia in supine position. In addition to the standard cervical transverse incision for coils insertion, a 5 cm skin incision was done vertically at the anterior axillary fold on the left side for battery placement. Anatomical dissection was carried out in both incisions to accomplish the implantation procedure [14], [15]. To ease and minimize the duration of coiling process we used two cervical nerve hooks to enable coils to wrap smoothly around the vagus nerve **(**Fig. 1**-B).**

#### Post-operative follow-up and device settings

Monitoring adverse events was done both before discharge and at regular post-operative visits. The device was turned on and programmed by the neurologist two weeks after surgery. Stimulation protocol was set with these parameters: current output is 0.25 mA, frequency 30 Hz, pulse width 500 µs, on-time 30 s, and off-time 5 min. The output current and the duty cycle were titrated with a gradually increased increment every 2 to 4 weeks according to the patient tolerance and the effect on seizure control. “Magnet stimulation” was offered to the patient and explained to the caregivers as an abortive therapy. All patients were followed at 3-month intervals until the last set follow-up interval at 12 months. After 6 months, if patients didn’t show at least 50 % seizure frequency reduction, rapid cycling mode was offered. After VNS implantation, ASMs dosages were kept unchanged to avoid bias of reducing seizure frequency or poor tolerance.

### Follow-up seizure response

Seizure frequency was noted at 12-month interval. Data was obtained from outpatient, inpatient charts, operative reports, and telephone calls. The overall response in seizure improvement was assessed by the percentage of reduction in total seizure frequency at 12 months in comparison to baseline seizure frequency in both groups.

### Cognitive assessment

For assessment of the cognitive functions, we adopted five cognitive domains of the Modified Mini-Mental State Examination (MMSE), including “orientation, attention-concentration, registration, recall and language” (Table 4) [16], [17]. It has been used effectively as a screening tool for cognitive deficits in children with cerebral palsy (CP) [18], epilepsy [19], Duchenne muscle dystrophy [20] since it is more practical and cost-effective than detailed neuropsychological testing that requires time and resources, such testing is more suitable for low middle income countries. The cognitive scores were calculated at baseline and 12 months of follow-up by completing the questionnaire through an interview with the caregivers for both groups. Baseline overall cognitive scores were calculated and compared initially for both groups to avoid selection bias.

### Statistical analysis

All statistical methods were conducted using SPSS version 19.0 (IBM, USA). Because of our small sample size, the statistical hypothesis was examined by the non-parametric tests. A descriptive analysis of all demographic and patient characteristics was presented by median and range. Pre- and post-treatment parameters were compared using the Wilcoxon sign rank test. All group-wise matching and comparative analyses were done using Chi-square for categorical variables and the Mann-Whitney *U* test for continuous variables. The significant threshold was 0.05 without the Bonferroni correction needed. Also, we conducted *t*-test for statistical sub-analysis of cerebral palsy patients in VNS group and compared it with BMT group.

### Ethical approval

The study was approved by the ethics committee of the Faculty of Medicine, Ain Shams University, and informed consent was obtained for participation by caregivers. Ethical approval number: FMASU R190/2023.

## Results

### Patient clinical characteristics

Seventeen patients were included in the study. Nine patients were in VNS group, and eight patients were in BMT group. Ten (58.8 %) were males and seven (41.1 %) were females. In the BMT group, the median age of presentation to our institute was 9.5 years (7–12) and the median age of onset of seizures was 0.75 years (0–2). In the VNS group, the median age on presentation to our institute was 10.5 years (7–17) and the median age of onset of seizures 9 months (3 months-11 years). The median age at VNS implantation was 10 (4–12) years. The median epilepsy duration was 8.5 (5.5–12) years in the BMT group and 8 (4–16.75) years in the VNS group. The median number of ASMs used was 3 (3–4) in the BMT group and 3 (3–4) in the VNS group. Detailed demographic and clinical characteristics of both groups were displayed in Table 1, Table 2**.** Thirteen children (76.4 %) had cerebral palsy. Four children had genetic epilepsy (23.5 %), two had tuberous sclerosis complex (TSC) (11.7 %), one had Lennox-Gastaut Syndrome (LGS) (5.9 %), and one had Focal Familial Epilepsy (5.9 %).

**Table 1:** Demographic data and clinical profile of children in the BMT group**. CBZ**: carbamazepine, **CP**: cerebral palsy, **GTC**: generalized tonic-clonic seizures, **LVT**: Levetiracetam, **OXC**: oxcarbazepine, **PHT**: Phenytoin, **VPA**: valproic acid, **GTC**: Generalized tonic-clonic, **M**: Myoclonic, **T**: tonic, **AT:** Atonic, **PC**: partial complex seizures.

**Patient**	**1**	**2**	**3**	**4**	**5**	**6**	**7**	**8**
**Age on presentation (years)**	11	7	9	7	12	10	7	11
**Biological Sex**	F	F	M	F	M	M	M	M
**Age at onset of seizures (months)**	24	18	12	6	birth	6	2	24
**Etiology**	Hemiplegic CP secondary to brain tumor	Post meningitic CP	Post anoxic CP	Post anoxic CP	Post anoxic CP	Post anoxic CP	Post anoxic CP	Post anoxic CP
**Seizure type**	PC	GTC, PC	AT	GTC, PC	GTC, M	PC	GTC	GTC, AT
**Baseline seizure frequency/month**	112	30	15	159	10	120	30	30
**Seizure frequency at 12 months/month**	84	30	60	75	10	40	10	60
**Baseline Seizure duration (minutes)**	60	50	120	90	180	90	120	90
**Seizure duration at 12 months (minutes)**	60	40	60	60	120	60	60	60
**Current ASMs**	VPA, CBZ, LVT	VPA, LVT	VPA, CBZ, LVT	VPA, LVT	PHT, LVT	OXC, LVT	CBZ, LVT	LVT
**EEG findings**	Left hemispheric focal epileptiform discharges	multifocal epileptiform discharges	Generalized epileptiform discharges	Generalized epileptiform discharges	Generalized epileptiform discharges	Temporal focal epileptiform discharges	Generalized epileptiform discharges	Generalized epileptiform discharges

**Table 2:** Demographic data and clinical profile of children in VNS-group **CP**: cerebral palsy, **GTC**: generalized tonic-clonic seizures, **LMT**: lamotrigine, **LVT**: Levetiracetam, **OXC**: oxcarbazepine, **PHT**: Phenytoin, **VPA**: valproic acid, **CBZ**: Carbamazepine, **TSC**: Tuberous Sclerosis Complex, **LGS**: Lennox-Gastaut Syndrome. **ADV**: Andovipamide, **GTC**: Generalized tonic-clonic, **M**: Myoclonic, **T**: tonic, **At**: Atonic, **PC**: partial complex seizures.

**Patient**	**1**	**2**	**3**	**4**	**5**	**6**	**7**	**8**	**9**
**Age on presentation (years)**	9	9	13	7	17	12	15	7	4
**Biological sex**	M	M	M	F	F	F	M	M	F
**Age at onset of seizures (months)**	birth	24	144	6	3	12	132	birth	24
**Age at implantation**	8	5	0.5	4	12	12	12	6	5
**Etiology**	post kernicterus CP	Familial focal epilepsy	Post anoxic CP	TSC	Post anoxic CP	Post anoxic CP	TSC	LGS	Post anoxic CP
**Seizure type**	GTC, T, M	GTC, PC	GTC	GTC, PC	GTC, PC	GTC, T	GTC, PC	GTC, PC, M, At	GTC, PC
**Baseline seizure frequency pre-VNS/month**	20	30	60	60	8	300	150	60	60
**Seizure frequency at 12 months post-VNS/month**	20	10	8	30	1	10	30	32	1
**Seizure duration before VNS (minutes)**	90	120	120	60	60	60	180	60	60
**Seizure duration after VNS (minutes)**	60	120	40	10	20	60	60	30	30
**Current ASMs**	LVT, VPA	LMCT, PHT	LVT, VPA	VPA, PHT, TPX	TPX, VPA, ADV	LVT, VPA	VPA, LVT, LAC	LVT, VPA	VAL, LVT, CBZ
**EEG findings**	Bitemporal and bifrontal epileptiform discharges	multifocal epileptiform discharges	multifocal epileptiform discharges	multifocal epileptiform discharges	multifocal epileptiform discharges	multifocal epileptiform discharges	Generalized epileptiform discharges	multifocal epileptiform discharges	multifocal epileptiform discharges

### Seizure response

The median seizure frequency was 30 (10–150) seizures/month at baseline and 50 (10–84) seizures/month at 12 months in the BMT group. Whereas the median seizure frequency was 60 (0.3–300) seizures/month pre-implantation and 9 (0.1–30) seizures/month at 12 months post-implantation in the VNS group. At 12 months of the follow-up period, seizure frequency showed reduction from baseline which was potentially trending among the VNS group (p = 0.018), and non-significant among the BMT group (p = 0.89). The overall rate of total seizure frequency reduction from baseline frequency of the BMT group was unchanged in 25 %, decreased by 25–49.9 % in 37.5 %, decreased by 50–74.9 % in 12.5 %, decreased by 75–90 % in 0 %, decreased by ˃ 90 % in 0 % and worsened in 25 %. In the VNS group, it was unchanged in 11.1 %, decreased by 25–49.9 % in 11.1 %, decreased by 50–74.9 % in 22.2 %, decreased by 75–90 % in 22.2 %, decreased by ˃ 90 % in 22.2 % and worsened in 0 % as shown in Table 3.

**Table 3:** Overall percentage reduction in seizure frequency at 12 months in both groups.

**Change in total seizure frequency at 12 months**	**BMT Group** **N (%)**	**VNS group** **N (%)**
Unchanged	2 (25 %)	1 (11.1 %)
Decreased by 25–––49.9 % of the baseline frequency	3 (37.5 %)	1 (11.1 %)
Decreased by 50–––74.9 % of the baseline frequency	1 (12.5 %)	3 (33.3 %)
Decreased by 75–––90 % of baseline frequency	0 (0 %)	2 (22.2 %)
Decreased by > 90 % of the baseline frequency	0 (0 %)	2 (22.2 %)
Worsened (increased by 16.67 % of baseline frequency)	2 (25 %)	0 (0 %)

### Effect on cognitive functions

**Fig. 2:**
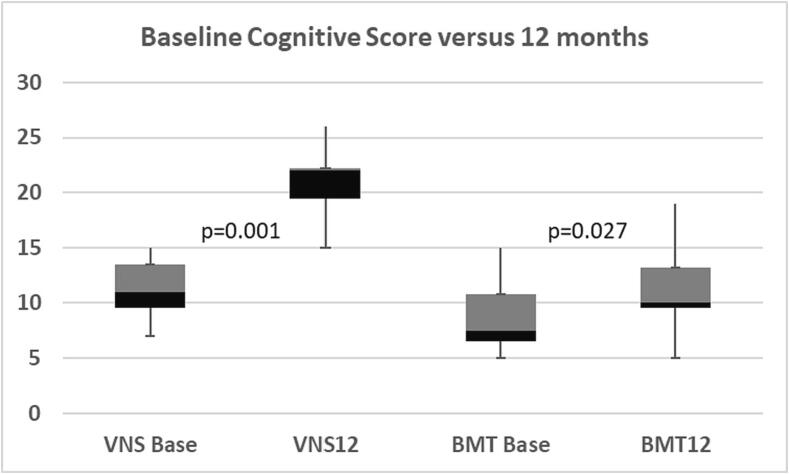
Chart showing mean cognitive score for VNS group and BMT group at baseline and 12 months follow up.

**Fig. 3:**
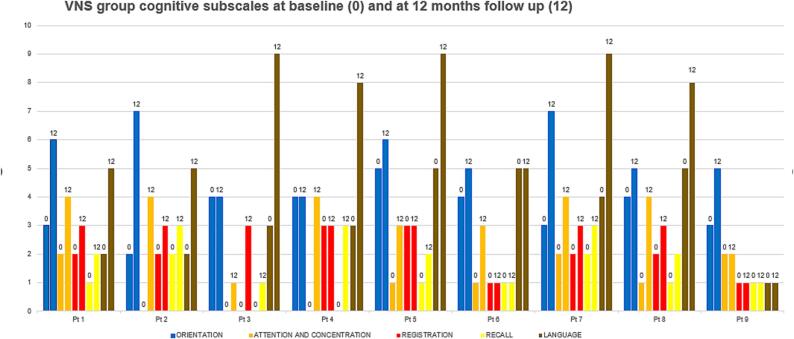
Chart showing subscales (color coded) of cognitive score for VNS group at baseline (0) and at 12 months follow up (12).

**Fig. 4:**
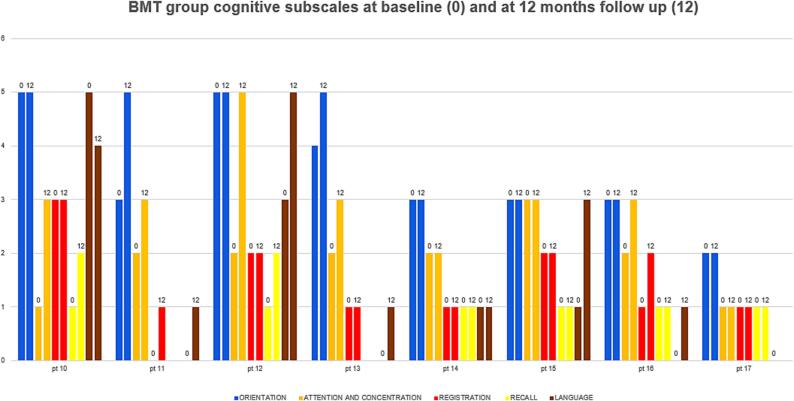
Chart showing subscales (color coded) of cognitive score for BMT group at baseline (0) and at 12-months follow-up (12).

Cognitive functions were assessed by using the MMSE for children. An average cognitive score was plotted for each child in each group at baseline and 12-month follow-up for the BMT group and at pre-implantation and 12-month post-implantation in the VNS group Table 4. The median overall cognitive score at baseline for BMT group was 7.5 (5–15) and for the VNS group was 11 (10–15). At 12 months for the BMT it was 10 (5–19) and for the VNS group it was 21 (15–26). Patients in both groups showed potentially trending improvement in their overall cognitive function scores at 12 months as compared to baseline scores (p = 0.027), (p = 0.012), respectively (Fig. 2). Baseline cognitive scores were comparable for both groups with no statistically significant difference (p = 0.44), whereas at 12 months, a statistically significant improvement was noted among the VNS group as compared to the BMT group (p = 0.001) (Fig. 2). The best outcome was observed in the attention and concentration and recall (subscale post-VNS implantation), respectively (p = 0.011, p = 0.011) as shown in Fig. 3 as compared to BMT group whose cognitive subscales were plotted in Fig. 4.

**Table 4:** Cognitive domains adopted from Modified Mini-Mental State Examination (MMSE) **(16)**.

**FUNCTION**	**TESTS**	**SCORE**
**ORIENTATION**	**Was the child able to:-** Say his/her name? **Yes (1) No (0)** Say his/her father’s name? **Yes (1) No (0)** Identify gender? **Yes (1) No (0)** Recognize parent? **Yes (1) No (0)** Identify today’s date? **Day (1)/ Date (1)/ Month (1)/ Year (1)** Recognize home? **Yes (1) No (0)** Recognize country? **Yes (1) No (0)** Recognize city? **Yes (1) No (0)** **Total score (__/11)**	**(__/11)**
**ATTENTION AND CONCENTRATION**	Was the child able to count from 1 to 4 **(__/4)** Was the child able to count backwards from 4 to 1**(__/4)** **(One point for each digit)** **Total score (__/8)**	**(__/8)**
**REGISTRATION**	**Was the child able to identify three objects?** Ball (__/1) Tree (__/1) Chair (__/1) **Total score (__/3)**	**(__/3)**
**RECALL**	**Was the child able to recall the three objects?** Ball (__/1) Tree (__/1) Chair (__/1) **Total score (/3)**	**(__/3)**
**LANGUAGE**	**Was the child able to:** Name a body part? (One point for each body part named) **(__/5)** Obey a three-step command: “Unwrap the toffee, give toffee to doctor, eat the toffee” **(One point for each step) (__/3)** Was the child able to repeat a sentence? **(__/1)** Can the child read his name? **(…/1)** Was the child able to write his name? **(__/1)** Was the child able to draw a circle? **(__/1)**	**(__/12)**
	**TOTAL SCORE**	**(__/37)**

#### VNS in cerebral palsy patients

**Fig. 5:**
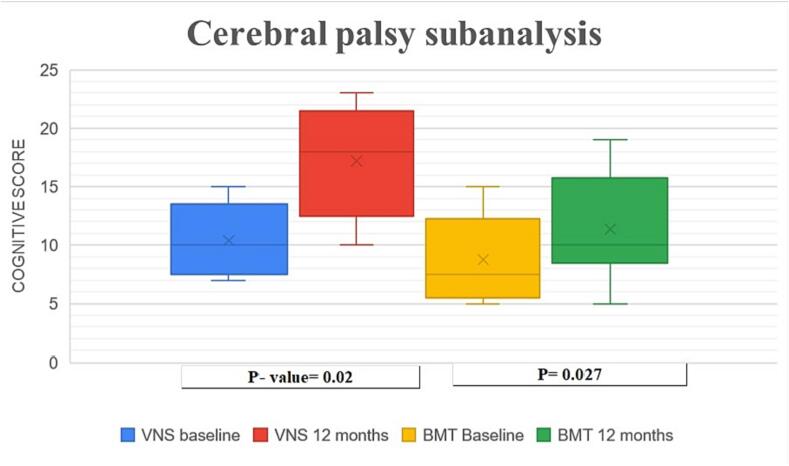
Chart showing mean cognitive score for VNS in cerebral palsy patients and BMT group at baseline and 12 months follow up.

We conducted a statistical sub-analysis on cerebral palsy patients who underwent VNS for seizure control as a part of the VNS group (VNS CP). Five children (patients 1,3,5,6,9) out of the VNS group had a diagnosis of cerebral palsy, in this sub-analysis a statistically significant improvement of cognitive functions was observed (p = 0.02). The BMT group (eight patients diagnosed with cerebral palsy) also showed statistically significant improvement of the cognitive subscales (p = 0.027). In a comparison between VNS CP patients and BMT group, the VNS group showed more significant improvement (Fig. 5).

Concerning the effect of seizure control on rehabilitation programs outcome, data retrieved from rehabilitation sheet scores of children submitted in our study was referring to 100 % regular attendance to rehabilitation sessions of children in VNS CP group (meaning no missed sessions due to frequent seizers), compared to 75 % attendance score in BMT group. This is in comparison to baseline data that revealed less than 50 % attendance score in both groups. Improving attendance score reflected positively on improvement of physical performance and motor learning in children with cerebral palsy.

#### VNS in genetic mutations

We reported two children with Tuberous Sclerosis, one boy child with Lennox Gastaut Syndrome (LGS) and one boy child with Familial Focal Epilepsy (FFE). All patients showed significant reduction in seizure frequency and cognitive improvement within the VNS group at 12-month records.

## Discussion

### Cognitive decline associated with epilepsy and IED

Approximately half of the patients with epilepsy have cognitive deficits at diagnosis [21]. More importantly, 26.4 % of children with epilepsy have suboptimal global cognitive development [22]. Hence, the International League Against Epilepsy (ILAE) Neuropsychology Task Force has recommended that all children with newly diagnosed epilepsy should undergo routine neuropsychological assessment to determine if they have subtle cognitive difficulties [23]. Various risk factors were directly correlated to cognitive deficits and behavioral problems such as the age of seizure onset, duration of epilepsy, and polytherapy, meaning that the earlier the onset, the longer the duration (≥5 years), and the higher the number of the ASMs the poorer the performance across all cognitive domains [24]. This is in agreement with our results where the median age of onset of seizures was 9 months in each group, and the median duration of epilepsy was between 8 and 8.8 years and the median number of ASMs was 3. It was recorded that all included children were previously on different categories of ASMs, and we noticed the effect on the interictal states induced by these medications. This could be explained by the enduring changes driven by multiple usage of ASMs on the brain function and structure that can affect the naturally occurring homeostatic seizure-suppressing mechanisms with adverse consequences on the normal neuronal function [25].

Epilepsy is frequently associated with interictal epileptiform discharges (IED’s) which have a dynamic occurrence in epilepsy, meaning they are paroxysmal and may not be captured in EEG [26]. Zeng et al. illustrated the pathophysiology of cognitive impairment due to epilepsy through experimenting different biochemical mechanisms that relate epilepsy to cognitive impairment [27]. A study by Bogaert et al., showed that IED cause glucose hypometabolism in neurons as was seen by positron emission tomography (PET) MRI during IED; they also pointed out that the abundance of IED during sleep could have a direct impact on memory consolidation and physiological neuroplasticity [27]. In our study, all children in both groups had multifocal IEDs, which we believe were at least partially responsible for some of their cognitive disability.

The question of whether the burden of IEDs in patients with epilepsy is related to cognitive deficits has been debated with conflicting opinions on whether to treat or not, especially in children. As early as in the 80 s, it has been hypothesized that paroxysmal IEDs are associated with mild transient cognitive deficits [28]. That can be neurophysiologically explained by disruption of long-term potentiation, which is the mainstay for the learning process [11]. Parisi et al. demonstrated that even apparently subclinical epileptic discharges (not only ‘subtle’ seizures) during sleep may disrupt cognitive functions because of sleep structure disruption [29]. More recently, both short nonconvulsive seizures and frequent epileptiform activity (>1% of the time) independent of the occurrence of seizures were found to affect global cognitive functioning with variable areas and magnitude [30]. This was supported by neurophysiological and functional neuroimaging evidence, highlighting that this may be related to either the short-term effects of IED on brain processing mechanisms, or the long-term effects due to prolonged distant inhibition of brain areas as well as impairment of the sleep-related learning consolidation processes [31]. Similarly, all our patients had poorly controlled seizures and abnormal EEG findings which explains their baseline poor cognitive scores.

These findings have encouraged the concept of implementing routine EEG recording in children even those with cerebral palsy to detect IEDs and initiate treatment in the absence of clinical epilepsy aiming at improving their prognosis and QOL [32], [33]. Interestingly, it has been reported that VNS had supplementary beneficial effects on behavioral aspects, particularly on mood, independent from the effect on seizure frequency when used for treating children with DRE [34]. In the literature, there are different opinions about the relation between seizure control and cognitive improvement. Some studies are in favor of early non-pharmacological management of DRE to achieve this goal, whereas other studies indicate no benefit in cognition even with seizure frequency reduction. Tsai et al., concluded that only seizure control was achieved rather than cognitive improvement [35]. On the other hand, Valova et al., addressed the effect of early treatment of DRE by non-pharmacological methods on improving cognitive function [23]. In addition, Knorr et al reported both cognitive improvement and quality of life improvement in children with post-encephalitic drug resistant epilepsy [36]. In our study, although limited by sample size, results are supportive of cognitive improvement after VNS implantation.

### Vagus nerve stimulation benefits

The effects of VNS can be divided into immediate and long-term. The immediate effect of VNS is more linked to seizure-control than cognitive outcome. A study by Yokoyama et al. [37] applied intraoperative electrocorticography during device implantation and recorded an immediate reduction in epileptic spikes. Several publications highlighted the possible effect of neurostimulation therapies such as VNS on improving mood, cognitive functions, sleep, and QOL in children with DRE [38], [39], [40]. One publication from the Middle East showed improvement in the QOL after VNS implantation in patients with partial seizures [8]. Our results were in agreement with these studies, where we found significant improvements in the cognitive scores of the VNS group at 12 months (post-implantation) as compared to their baseline (pre-implantation) scores. Moreover, this post-implantation cognitive scores improvement in the VNS group was found to be significantly higher when compared to the cognitive scores of the BMT group at 12 months. This improvement in the VNS group cognitive scores could be directly related to better seizure control, where 75 % of patients showed ≥ 50 % seizure frequency reduction and indirectly related to the proposed suppression of IEDs which is widely accepted as an established combination of behavioral disruption and neurocognitive impairment. A hospital-based study aimed at exploring the long-term role of VNS in 15 children with intractable seizures and described a long-term benefit at 9-month follow up [41]. In contrary, a recent report in 2022 highlighted the delayed improvement in memory with either invasive or non-invasive VNS therapy [42].

### Epilepsy and cerebral palsy

It is worth mentioning that 76.4 % of children in our study had cerebral palsy, which means that cognitive improvement was observed even in children with developmental disabilities and underlying structural brain pathology. This is in line with the previously proposed treatment protocol by Jaseja [11] that recommends VNS implantation for the treatment of DRE in children with cerebral palsy to improve their neurocognitive deficits and QOL through its dual anti-epileptic and IED-suppression therapeutic mechanism. Also, Jaseja et al. proposed inclusion of EEG in the management protocol of CP patients and treatment of IEDs even in the absence of clinical seizures for better cognitive outcome [42]. Ngugi’s study is important because illustrates a method to screen the neuropsychiatric burden of epilepsy in a low/middle income country [43]. Another promising study reported that VNS has a positive role in modifying autistic behaviors in younger children with autism and DRE regardless of seizure control and in the absence of training interventions [44]. In contrast to our results, Tsai and his colleagues did not find any significant improvement in cognitive functions but mainly reduced seizure frequency and parent–child related stress [36]. We believe that the promising effects of VNS on cognitive functions are multifactorial; due to seizure frequency reduction [45], reduction of polytherapy with its established cognitive and behavioral hazards, and the improvement in the quality of sleep through reduction of IEDs especially in Rapid Eye Movement (REM) and delta sleep [46]. This might be pathophysiologically explained by the effect of VNS on the acute surge of norepinephrine in the prefrontal cortex and hippocampus with the resultant seizure reduction and its role in long-term potentiation in the dentate gyrus [47] that in turn enhances learning, memory, and mood.

### Genetic mutations associated epilepsy

There is paucity of evidence in the literature that mention the role of VNS in genetic epilepsies other than FCD, LGS, Tuberous Sclerosis and Dravet Syndrome (DS) [48]. In our series, the child with FFE showed a significant seizure reduction and cognitive improvement after VNS implantation.

In agreement with Parain et. al. who reported a multicenter study of 10 tuberous sclerosis patients, [49] there was a significant improvement of cognitive subscales and seizure reduction in our two children presented with DRE due to tuberous sclerosis. Also, in concordance with a *meta*-analysis on 480 LGS cases treated with VNS which reported 54 % seizure responders and cognitive improvement, [50] in our series the child with LGS showed obvious improvement with high parent satisfaction.

### Early versus late VNS implantation in DRE

There is reason to believe that earlier treatment with non-pharmacological modalities will yield better cognitive functions in such children. Some reports in the literature addressed more improvement in cognitive domains in children < 6 years than those between the age of 6–14 years [51]. Other reports show no improvement of cognitive functions [52]. These authors believe that in order to answer this question, a controlled study with a large sample size and homogenous epilepsy etiology, and wide age difference should be conducted.

### Anti-seizure medication usage in both groups

In our study, all patients were on at least two ASMs except for a single patient in the BMT group. Patients continued on ASMs post VNS implantation and had improved seizure control. Most of the patients in our study were on levetiracetam (LVT), 6 patients in VNS group and all patients in BMT group. Several studies have been published on the long-term use and retention rates of levetiracetam in children with refractory epilepsy, most of which showed promising data about the tolerability and safety of levetiracetam in children [53]. Verrotti A. et al. described in their study that LVT showed minimal behavioral adverse effects and non-statistically significant effect on cognitive functions with long term use [54].

## Conclusion

To our knowledge, this is the first comparative retrospective pilot study from our region suggesting a potential role of VNS in improving cognitive functions as compared to the BMT group by using a cost-effective screening tool, with a significant effect observed in cerebral palsy patients. This is of great importance, especially in limited-resources countries as VNS has shown good safety profile, added value to cognitive function, and additive effect to seizure control which can be of high economic value in the long term.

## Study limitations

The small sample size, the heterogeneity of pathologies causing epilepsy may be a cause of confounding bias for results and the lack of randomization are the main limitations in our study.

The long term use of ASMs on cognitive function can’t be excluded. However, we believe that although the study’s sample is small, it could help to set the basis for large-scale prospective long-term studies.

## Financial & funding statement

No financial relations between authors and co-authors. This research did not receive any specific grant from funding agencies in the public, commercial, or not-for-profit sectors.

## CRediT authorship contribution statement

**Mohamed Ashraf Mahmoud:** Writing – original draft, Formal analysis, Data curation, Conceptualization. **Omnia El Rashidi:** Formal analysis, Conceptualization. **George Halim:** Formal analysis, Data curation. **Mohamed Amgad Elkholy:** Writing – original draft, Formal analysis, Data curation, Conceptualization. **Osama Aglan:** Formal analysis, Data curation. **Abdel Rahman El Sabbagh:** Formal analysis, Data curation. **Ahmed Kamel Basha:** Formal analysis, Data curation. **Hussein Hamdi:** Formal analysis, Data curation. **Ahmed M. El Sayed:** Formal analysis, Data curation. **Dina Amin Saleh:** Formal analysis, Data curation, Conceptualization. **R.H. Shatla:** Formal analysis, Data curation. **Walid Abdel Ghany:** Supervision, Project administration, Methodology, Conceptualization.

## Declaration of competing interest

The authors declare the following financial interests/personal relationships which may be considered as potential competing interests: Mohamed Amgad Elsayed Elkholy reports administrative support was provided by Ain Shams University Faculty of Medicine. Mohamed Amgad Elsayed Elkholy reports a relationship with Ain Shams University Faculty of Medicine that includes: employment and non-financial support. If there are other authors, they declare that they have no known competing financial interests or personal relationships that could have appeared to influence the work reported in this paper.
